# Analysis of Changes in Sleep Quality and Patterns after Hip Fracture Using Real Evidence of Artificial Intelligence Linked (REAL) Hip Cohort Data

**DOI:** 10.3390/medicina59122125

**Published:** 2023-12-05

**Authors:** Yonghan Cha, Jung-Taek Kim, Jin-Woo Kim, Jin-Gu Lee, Sang-Yeob Lee, Hyun-Bin Kim, Yang Jae Kang, Won-Sik Choy, Jun-Il Yoo

**Affiliations:** 1 Department of Orthopaedic Surgery, Daejeon Eulji Medical Center, Eulji University School of Medicine, Daejeon 35233, Republic of Korea; 2 Department of Orthopedic Surgery, Ajou University School of Medicine, Ajou Medical Center, Suwon 16499, Republic of Korea; 3 Department of Orthopaedic Surgery, Nowon Eulji Medical Center, Eulji University School of Medicine, Seoul 01830, Republic of Korea; 4 Department of Psychiatry, Daejeon Hospital of Korea Worker’s Compensation and Welfare Service, Daejeon 34384, Republic of Korea; 5 Department of Biomedical Research Institute, Gyeongsang National University Hospital, Jinju 52727, Republic of Korea; 6 Department of Biomedical Research Institute, Inha University Hospital, Incheon 22332, Republic of Korea; gusqls2463@naver.com; 7 Division of Life Science Department, Gyeongsang National University, Jinju 52828, Republic of Korea; 8 Department of Orthopaedic Surgery, Inha University Hospital, Incheon 22332, Republic of Korea

**Keywords:** elderly, hip fracture, post-traumatic distress syndrome, post-traumatic diagnosis scale

## Abstract

*Background and Objectives*: Hip fractures are commonly found in elderly patients, and often result in chronic pain and decreased physical function, as well as worsening of overall health. It is known that early surgical intervention during the acute phase and rehabilitation are important for improving clinical outcomes for these patients. However, the importance of management for improving the quality of life of these patients is becoming more emphasized. Studies on changes in sleep patterns after hip fractures are rare overseas. Therefore, the aim of this study is to investigate the prevalence of sleep disturbance in patients with hip fractures and to analyze the changes in sleep disturbance after surgery by comparing the preoperative and postoperative results. *Materials and Methods*: During the period from August 2022 to January 2023, patients who underwent surgical treatment for hip fractures and were recruited into the REAL Hip Cohort were selected as research subjects. The sleep survey was conducted using the Pittsburgh Sleep Quality Index (PSQI). The PSQI is composed of 18 questions, each divided into areas of sleep quality, sleep latency, duration, efficiency, disturbance, use of medication, and daytime dysfunction. Each area is scored 0–3 points and the total is 0–21. A score greater than five indicates sleep disorder. The PSQI was surveyed during hospitalization and three months after surgery for post-fracture sleep status. To analyze changes before and after the fracture, paired T-tests and chi-square tests were performed. *Results*: From August 2022 to January 2023, a total of 40 patients who were recruited into the REAL Hip Cohort responded to the PSQI survey. The average age was 77.4 years and 36 were female. Sleep quality worsened from 0.75 ± 1.0 before surgery to 1.4 ± 1.0 three months after surgery (*p* = 0.019), and sleep efficiency also worsened from 0.4 ± 0.6 to 1.4 ± 1.0 (*p* < 0.001). The PSQI increased from an average of 5.2 ± 2.8 before surgery to 8.2 ± 4.2 three months after surgery (*p* = 0.007), and the number of patients who could be diagnosed with sleep disorders also increased from 12 (40%) to 24 (60%) (*p* = 0.030). *Conclusions*: A decline in overall sleep status was observed in patients in a survey on sleep patterns three months after hip fracture. Additional management is needed to improve their sleep patterns.

## 1. Introduction

Elderly hip-fracture patients face a concerning trend in their incidence worldwide [1]. The global incidence of hip fractures among the elderly population has been steadily increasing, primarily due to the aging demographics in many countries [2]. This rise is indicative of the challenges associated with an aging society, as osteoporosis and falls become more prevalent. This increasing incidence is closely linked to higher mortality rates among elderly hip-fracture patients. The frailty and comorbidities common in this age group make the management of these fractures particularly complex, and as a result, mortality rates remain alarmingly high [2]. Efforts to address this issue should not only focus on treatment but also on preventive measures and improving overall healthcare for the elderly population to mitigate the impact of hip fractures and reduce the associated mortality rates [3].

Elderly populations are prone to sleep disorders due to changes in sleep duration and patterns [4]. It is considered a normal part of aging that sleep time decreases as one ages from infancy to adulthood and into old age. Nevertheless, the prevalence of elderly people complaining of sleep disorders is known to be high, at around 20–30% [5]. In elderly patients with hip fractures, delirium is one of the most common complications that can occur, and it is caused by changes in normal neural activity [6]. Nighttime delirium symptoms can cause disturbances in sleep and wake cycles, as well as alter circadian rhythms [7]. The secretion of the pineal hormone melatonin, which is important for maintaining circadian rhythm, can be affected not only by light, temperature, and activity but also by surgery, anesthesia, and medication [8]. Consequently, we believe that elderly patients with hip fractures may frequently experience sleep disorders.

However, few studies have shown the relationship between hip fractures and sleep disorders. Berry et al. reported that the use of benzodiazepines increased the risk of hip fractures in patients over 50 years of age who reside in nursing homes [9]. Furthermore, Yoo et al. reported that the use of benzodiazepine after surgery in patients over 65 years old with hip fractures increased all-cause mortality [10]. Kuo et al. investigated sleep duration in elderly patients with hip fractures and diabetes and reported that 78% of them had abnormal sleep duration [11]. Parimi et al. conducted a study on sleep disturbance in patients who underwent total hip arthroplasty (THA) due to hip osteoarthritis (OA) [12]. However, these studies focus on the side effects of sleep aids or sleep duration, and there is a lack of analysis on sleep quality. Moreover, the study only focuses on diabetic patients or has a diverse group of patients included.

Elderly patients with hip fractures often experience chronic pain and physical dysfunction as well as the deterioration of health after the fracture, resulting in a decrease in their quality of life [13]. It is known that early surgical treatment and rehabilitation during the acute phase is important to improve the clinical outcomes of these patients, as these elderly patients with hip fractures often exhibit chronic pain and decreased physical function, leading to a decline in their quality of life [2].

Therefore, the aim of this study is to investigate the prevalence of sleep disturbance in patients with hip fractures and to analyze the changes in sleep disturbance after surgery by comparing the preoperative and postoperative results.

## 2. Materials and Methods

### 2.1. Ethics Statement

The protocol of our study was approved by our institutional review board (IRB No. EMC2021-12-002-001) and registered with the Clinical Research Information Service (Registration No. KCT0007374).

### 2.2. Data and Participants

The Real Evidence of Artificial intelligence Linked Hip fracture Cohort (REAL Hip Cohort) was constructed using data from 4 university hospitals in August 2022 [14]. The purpose of this cohort is to develop medical and surgical treatment guidelines for elderly patients with hip fractures based on real-world data. The subjects of this REAL Hip Cohort were patients aged 50 years or older with hip fractures who visited one of 4 university hospitals. The data were prospectively collected using an android-based application (Doctor logs, Deevo, Republic of Korea) and stored in a database (DMS, Deevo, Republic of Korea), and these data were used for image-data analysis using artificial intelligence, as well as for analysis research on evidence for the clinical management and evaluation of hip fractures. The collected data included demographic factors such as age, sex, area of residence, cohabitants, and underlying disease, as well as laboratory data, radiographs, surgery-related factors, and patient-reported outcomes conducted during hospitalization. In addition, regular outpatient follow-ups were conducted at 6 weeks, 3 months, 6 months, and 12 months after surgery or injury, and data were collected at each period.

Changes in sleep patterns were investigated using the Korean version of the Pittsburgh Sleep Quality Index (PSQI) from August 2022 to January 2023 in the REAL Hip Cohort [15,16]. Surveys were conducted twice, during hospitalization and 3 months after surgery. The survey conducted during hospitalization investigated sleep patterns before fracture. The PSQI consists of 18 items measuring 7 components, including sleep quality, sleep latency, sleep duration, sleep efficiency, sleep disturbance, use of sleep medication, and daytime dysfunction of sleep based on the previous month. Each component is scored from 0 points to 3 points, and the total PSQI score is calculated as the sum of the 7 components with possible scores ranging from 0 points to 21 points. A PSQI score of > 5 was considered to indicate poor sleep quality in our study [15,16]. Those who did not respond to the survey, and patients who were unable to take the survey due to future cognitive function were excluded from the study. The inclusion and exclusion criteria are as follows.

Inclusion criteria:
  (1) age ≥ 65 years;
  (2) diagnosis: neck, intertrochanter, and subtrochanter fractures;
  (3) surgical treatment, such as internal fixation or arthroplasty.

Exclusion criteria:
  (1) cognitive impairment or dementia;
  (2) incomplete medical records;
  (3) refusal to consent.

## 3. Statistical Analysis

Preoperative factors, encompassing demographic data such as age, sex, body mass index, preoperative Koval grade, and medical comorbidities assessed using the modified Charlson’s comorbidity index (CCI) [17], along with injury timing (day/night), right/left side, fracture site (femoral neck, intertrochanteric fracture, or subtrochanteric fracture), presence of atypical fracture, indoor or outdoor injury, associated fractures, past history of fractures, residential area (metropolitan or non-metropolitan), living arrangements with family, and preoperative assessments using the EuroQol-5 dimension (EQ-5D), Harris Hip Score (HHS), and Western Ontario and McMaster Universities Osteoarthritis Index (WOMAC) score, were examined.

Surgery-related factors, including the type of surgery, operation duration, anesthesia type (general or spinal), transfusion requirements, and peri-articular injection for pain control, were also analyzed.

Postoperative considerations incorporated postoperative opioid use, occurrences of postoperative delirium and medical complications, venous thromboembolism prophylaxis, and clinical parameters at the 3-month postoperative mark, such as the place of residence (hospital or home), Koval grade, EQ-5D, WOMAC, and HHS.

For the analysis of our data, we applied chi-square tests to assess categorical variables, while paired T-tests were employed to evaluate numerical variables. The significance was determined by considering all *p* values less than 0.05 to be statistically significant. The statistical analysis was conducted using IBM SPSS Statistics version 20.0 (IBM, Chicago, IL, USA).

## 4. Results

A total of 40 patients, who responded to the PSQI questionnaire, were included in our study (Table 1). The average age of the participants was 77.4 ± 10.3 years, and there were 35 women (90%). Among all patients, 55% had intertrochanteric fractures, 25% had neck fractures, and the remaining cases involved subtrochanteric fractures. The average CCI was 3.8 ± 1.6. The EQ-5D before surgery was 5.85 ± 1.2, and the HHS before surgery was 83.6 ± 9.4. Thirty-two patients underwent internal fixation, and eight patients underwent hemiarthroplasty. Delirium occurred in six patients (15%) after surgery.

The preoperative PSQI score averaged 5.2 ± 2.8, and it showed a significant increase to 8.2 ± 4.2 after a 3-month post-surgery period (*p* = 0.007). The percentage of patients with a PSQI score exceeding five also rose from 40% (12 patients) before the injury to 60% (24 patients) after the surgical intervention (*p* = 0.030). Regarding the PSQI sub-scales, the score for sleep quality elevated from 0.75 ± 1.0 pre-injury to 1.4 ± 1.0 at the 3-month post-surgery mark (*p* = 0.019). Additionally, sleep efficiency exhibited a noteworthy increase from 0.4 ± 0.6 to 1.4 ± 1.0 (*p* < 0.001) (Table 2).

In the detailed items of the survey, the ability to fall asleep within 30 min increased from 1.0 ± 1.2 before surgery to 1.8 ± 1.3 three months after surgery, indicating an increase in difficulty falling asleep (*p* = 0.014). The frequency of waking up in the middle of the night or early morning to go to the bathroom increased (*p* = 0.017), as well as the frequency of waking up in the middle of the night or early morning (*p* = 0.006) (Table 3).

## 5. Discussion

The main results of the study indicated that the PSQI scores of individuals with hip fractures worsened after surgery in comparison to their scores prior to the surgery. The frequency of sleep disruption increased from 40% to 60% postoperatively after a period of three months, accompanied by a pronounced decrease in the subjective evaluation of sleep quality and efficiency. This was predominantly due to the difficulties in sleep onset and recurrent awakenings during the nocturnal hours for bathroom trips.

Epidemiologic studies on adult populations report that the prevalence of sleep disorders ranges from 14% to around 35% [15,18]. Studies have reported a high prevalence of sleep disorders in the elderly population, with rates as high as 62%. One research investigation examining the effect of sleep deprivation in 62 hip-fracture patients found that their average daily sleep duration was between 5 to 6.5 h [19]. However, the study was limited in its ability to diagnose specific sleep disorders or assess the quality of sleep. The majority of research exploring the relationship between sleep disruption and fractures focuses on the increased risk of fracture due to sleep disturbance, while the prevalence and deterioration of sleep disturbance and quality in hip-fracture patients remains underinvestigated [20,21]. In our investigation, 40% of individuals with hip fractures displayed symptoms of sleep disturbance prior to their fracture, which increased to 60% within three months post-fracture. The majority of the reported sleep disturbance was characterized by a decline in sleep quality and efficiency. To the best of our knowledge, this report represents the first analysis of the prevalence of sleep disturbance before and after a hip fracture.

Parimi et al. conducted a study examining sleep disturbance and quality in patients with hip osteoarthritis (OA) and accompanying hip pain [12]. They found that patients with hip OA displayed a higher degree of sleep fragmentation compared to those without, with this phenomenon being particularly pronounced in women experiencing severe hip pain. Sleep fragmentation was found to result from hip pain during a lying position, which was not related to other activities such as walking or climbing stairs. The authors suggested that the primary cause of this type of hip pain was inflammation such as OA or trochanteric bursitis. In contrast, in our study, hip-fracture patients did not exhibit sleep disturbance primarily due to pain. Instead, difficulties with sleep onset and maintenance were the main causes of sleep disorders. Internal fixation surgery, performed to stabilize the fracture site, typically requires three months for a hip fracture to heal. Surgical complications, such as implant irritation, non-union, and prosthesis loosening, have been associated with increased pain during motion, whereas pain at-rest and in a lying position seemed to be less implicated [22]. Therefore, pain control may be important for treating sleep disorders in these patients, but the focus of the treatment should be on starting and maintaining sleep.

Circadian disruption caused by sleep disturbance can decrease osteoblast function and promote bone resorption, which can increase the risk of fractures by impairing the repair mechanism [23,24]. Solving sleep disturbance can not only improve the quality of life for patients but also be important for bone health [25]. Benzodiazepine is one of the most representative hypnotics that can be used to solve sleep disturbance [9]. This drug is effective in promoting sleep initiation and maintenance, but its side effects, such as cognitive or memory impairment, rapid tolerance, rebound insomnia upon discontinuation, and substantial risk of abuse and dependence, can cause serious problems [26]. In our study, patients experienced difficulty falling asleep and maintaining sleep. Therefore, benzodiazepines seem to be effective medications for hip-fracture patients experiencing sleep disturbances. However, if elderly patients use benzodiazepines, the risk of falling increases, leading to an elevated risk of hip fracture [9]. If hip-fracture patients who did not use benzodiazepines before the fracture start to use them after the fracture, all-cause mortality may increase [10]. Z-drugs, such as zolpidem, zopiclone, and zaleplon, can reduce the risk of drug dependence, but the dose-dependent increase in fracture risk may still be a problem [27]. Additionally, Berry et al. reported that the risk of hip fractures in nonbenzodiazepine hypnotic drug users increased by 1.66 times, and it increased by more than 2 times depending on the severity of comorbid conditions or functional impairment. [9] Melatonin levels are at their highest between 1:00 a.m. and 4:00 a.m., and low nocturnal melatonin levels have been reported in delirium patients [8]. Delirium is considered to be accompanied by disturbances in sleep and wake cycles and circadian rhythm disturbances, so melatonin supplementation is expected to solve both delirium and sleep disturbance [8]. There have been reports that melatonin slightly improves sleep onset and sleep duration, but the magnitude of its effects varies, and its impact on elderly patients appears to be unverified. Therefore, we must consider the characteristics of sleep disturbance in the selection of hypnotics for elderly patients with hip fractures. And drugs need to be carefully chosen based on their efficacy and potential side effects in elderly patients with hip fractures.

Our study has several limitations. First, the number of patients recruited to our study was too small to rule out selection bias. We were unable to include many patients in the study because a significant number of them hesitated to address questions regarding psychiatric disorders. In this study, the required sample size was determined as 54 using G Power analysis, considering an effect size of 0.5, a significance level of 0.05, and a power of 95%. Therefore, the results of our study necessitate careful interpretation. In addition, the results of our study are considered to be underestimated because many patients with cognitive impairment, dementia, or comorbidity could have been excluded. Patients with cognitive impairment, despite the improvement of delirium in the course of treatment for hip fractures, were excluded from the study as they demonstrated a limited understanding of questions or were uncooperative. Additionally, patients admitted to the intensive care unit or receiving ventilator care were not eligible for the survey. Second, we were unable to examine certain factors contributing to sleep disorders, such as postoperative ambulation ability and the use of hypnotics. Due to the limited number of patients included in the study, conducting additional statistical analyses for risk factors was not feasible. Patients with femoroacetabular impingement are at a high risk of poor sleep quality, with a reported prevalence of sleep disturbance reaching 94% [28]. However, hip arthroscopic surgery has been shown to improve sleep quality. Similar observations have been noted in patients undergoing procedures such as rotator cuff repair, hip, and knee arthroplasty [29,30]. While adequate sleep is known to play a crucial role in postoperative healing, favorable clinical results from appropriate surgery can also impact postoperative sleep quality [31]. Nevertheless, there is limited clinical evidence establishing a clear relationship between postoperative positive outcomes and sleep quality. The use of hypnotics can be a crucial factor in improving sleep disorders, and unfortunately lacked thorough investigation in our study. However, when comparing sleep disturbances before and after hip fractures, an increase was observed following the hip fracture. Considering that hypnotics cannot entirely alleviate all forms of sleep disturbance, it is believed that our study’s crucial finding—that hip fractures increase the risk of sleep disturbance—has been underestimated. Further additional research is needed to complement the limitations of our study.

In conclusion, it has been observed that there is an increase in the prevalence of sleep disturbance among elderly patients after a hip fracture. Sleep disturbance is mainly due to the deterioration of sleep quality and efficiency. Efforts are needed to solve sleep disturbance in order to improve the quality of life and health of patients with hip fractures.

## Figures and Tables

**Table 1:** Demographics for included patients.

Variables	Total of 40 Patients
**Preoperative factors**	
Age (mean ± SD)	77.4 ± 10.3
Sex (women:men) (*n*, %)	36 (90%):4 (10%)
Body mass index (Kg/m^2^, mean ± SD)	22.7 ± 3.9
Preoperative Koval grade (mean ± SD)	1.3 ± 0.6
CCI (mean ± SD)	3.8 ± 1.6
Left:right (*n*, %)	8 (20%):32 (80%)
Fracture site (neck:intertrochaner/subtrochanter)	10 (25%):22 (55%):8 (20%)
Living with family (*n*, %)	38 (95%)
Pre-injury EQ-5D (mean ± SD)	5.85 ± 1.2
Pre-injury HHS (mean ± SD)	83.6 ± 9.4
Pre-injury WOMAC pain (mean ± SD)	2.5 ± 4.8
Pre-injury WOMAC stiffness (mean ± SD)	0.6 ± 1.6
Pre-injury WOMAC physical function (mean ± SD)	8.4 ± 15.7
**Surgery-related factors**	
Time from admission to surgery(days, mean ± SD)	3.6 ± 5.6
Surgery type (IF:BHA) (*n*, %)	32 (80%):8 (20%)
Anesthesia (general:spinal) (*n*, %)	38 (95%):2 (5)%
Operation time (minute, mean ± SD)	39.6 ± 23.2
Transfusion (mL, mean ± SD)	40 ± 123.1
**In-hospital factors**	
Postoperative delirium (*n*, %)	6 (15%)
Postoperative medical complications (*n*, %)	24 (60%)
Use of postoperative opioid (*n*, %)	10 (25%)
**Clinical factors at 3 months**	
Postoperative residential area (home:hospital:nursing home) (*n*, %)	30 (75%):8 (20%):2 (5%)
Koval grade at 3 months (mean ± SD)	3.02 ± 2.4
EQ-5D at 3 months (mean ± SD)	9.62 ± 2.2
HHS at 3 months (mean ± SD)	57.62 ± 19.7
WOMAC pain at 3 months (mean ± SD)	7.5 ± 6.3
WOMAC stiffness at 3 months (mean ± SD)	2.5 ± 2.4
WOMAC physical function at 3 months (mean ± SD)	36.7 ± 21.0

*n*; number, SD; standard deviation, IF; internal fixation, BHA; bipolar hemiarthroplasty, EQ-5D; EuroQol-5 dimension, HHS; Harris Hip Score, WOMAC; Western Ontario and McMaster Universities Osteoarthritis Index.

**Table 2:** Sub-scales of the Pittsburgh sleep quality index in hip-fracture patients.

Variables	Pre-Injury	3 Months After Operation	*p*-Value
Sleep quality (mean ± SD)	0.75 ± 1.0	1.4 ± 1.0	0.019
Sleep latency (mean ± SD)	1.4 ± 0.6	1.85 ± 1.0	0.070
Sleep duration (mean ± SD)	1.1 ± 0.9	1.4 ± 1.3	0.267
Sleep efficiency (mean ± SD)	0.4 ± 0.6	1.4 ± 1.0	<0.001
Sleep disturbance (mean ± SD)	1.2 ± 0.4	1.2 ± 0.4	1.000
Sleep medication (mean ± SD)	0.2 ± 0.7	0.2 ± 0.7	0.825
Daytime sleep dysfunction (mean ± SD)	0.8 ± 1.5	0.3 ± 0.6	0.094
Global score (mean ± SD)	5.2 ± 2.8	8.2 ± 4.2	0.007
Sleep problem (number, %)	12 (40%)	24 (60%)	0.030

SD, standard deviation.

**Table 3:** Comparison of sleep patterns and qualities using the Pittsburgh Sleep Quality Index between preoperative hip-fracture patients and 3-month-postoperative hip-fracture patients.

Variables	Pre-Injury Period	3 Months After Operation	*p*-Value
During the past month, what time have you usually gone to bed at night?	10:17 p.m. ± 90 min	10:00 p.m. ± 114 min	0.827
During the past month, how long has it usually taken you to fall asleep each night? (minute, ±SD)	35.5 ± 11.0	45.0 ± 30.4	0.240
During the past month, what time have you usually gotten up in the morning?	5:36 a.m. ± 114 min	6:24 a.m. ± 90 min	0.063
During the past month, how many hours of actual sleep did you get at night? (hour, mean ± SD)	6.7 ± 1.4	6.3 ± 1.9	0.383
Cannot get to sleep within 30 min (mean ± SD)	1.0 ± 1.2	1.8 ± 1.3	0.014
Wake up in the middle of the night or early morning (mean ± SD)	2.2 ± 0.9	2.8 ± 0.7	0.006
Have to get up to use the bathroom (mean ± SD)	2.0 ± 1.0	2.6 ± 1.0	0.017
Cannot breathe comfortably (mean ± SD)	0.1 ± 0.4	0.1 ± 0.4	1.000
Cough or snore loudly (mean ± SD)	0.2 ± 0.7	0.2 ± 0.6	1.000
Feel too cold (mean ± SD)	0.6 ± 1.0	0.6 ± 1.0	1.000
Feel too hot (mean ± SD)	0.1 ± 0.3	0.1 ± 0.2	0.577
Have bad dreams (mean ± SD)	0.3 ± 0.6	0.1 ± 0.2	0.163
Have pain (mean ± SD)	0.4 ± 0.7	0.5 ± 0.8	0.330
Other reason, including how often you have had trouble sleeping (mean ± SD)	0.7 ± 1.2	1.5 ± 1.5	0.040
During the past month, how often have you taken medicine to help you sleep? (mean ± SD)	0.2 ± 0.7	0.2 ± 0.7	0.825
During the past month, how often have you had trouble staying awake during social activity? (mean ± SD)	0.2 ± 0.5	0.5 ± 0.9	0.204
During the past month, how much of a problem has it been for you to keep up enough enthusiasm to get things done? (mean ± SD)	0.2 ± 0.5	0.4 ± 0.8	0.453
During the past month, how would you rate your sleep quality overall? (mean ± SD)	0.8 ± 1.0	1.4 ± 1.0	0.019

SD, standard deviation.

## Data Availability

Data is unavailable due to ethical restrictions.
